# Combined Novel Microfocused Ultrasound and Microneedle Fractional Radiofrequency System for Multilayered Facial Rejuvenation: A Prospective, Randomized, and Split‐Face Study

**DOI:** 10.1111/jocd.70455

**Published:** 2025-09-22

**Authors:** Ruiyao Wang, Guangling Peng, Yangmei Chen, Xinyi Shao, Lin Liu, Tingqiao Chen, Mengcen Shi, Judan Zhong, Yi Ou, Jin Chen

**Affiliations:** ^1^ Department of Dermatology The First Affiliated Hospital of Chongqing Medical University Chongqing China; ^2^ Department of Dermatology The Fifth People's Hospital of Chongqing Chongqing China; ^3^ Department of Dermatology Jiangjin District Maternal and Child Health Hospital of Chongqing Chongqing China

**Keywords:** clinical research, facial rejuvenation, microfocused ultrasound, microneedle fractional radiofrequency

## Abstract

**Background:**

With the aging population, the demand for skin anti‐aging treatments has been steadily rising, prompting the development of advanced non‐invasive therapies.

**Aims:**

To evaluate the efficacy and safety of microfocused ultrasound (MFU) combined with microneedle fractional radiofrequency (MFR) for facial rejuvenation.

**Methods:**

This study involved 26 patients experiencing facial laxity. Each patient received one full‐face MFU treatment and one MFR treatment on one side of the face, which was randomly assigned. All treatments were performed on the same day. Facial photoaging parameters were assessed using VISIA, skin fat thickness changes were measured via ultrasound, and subjective evaluations were recorded at Months 0, 1, and 3. Side effects were recorded during treatments and each follow‐up visit.

**Results:**

The VISIA analysis demonstrated notable enhancements in skin texture, wrinkles, pores, spots, and red areas on the combined treatment side. Ultrasound examinations revealed a significant reduction in subcutaneous fat thickness, particularly at the masseter and the middle of the cheek on the combined side. The study showed improvements in the Global Aesthetic Improvement Scale (GAIS) and Wrinkle Severity Rating Scale (WSRS) scores, with the combined treatment side outperforming the control side at the 3 month mark. Over 90% of participants expressed satisfaction with the treatment outcomes. Mild side effects, such as erythema, purpura, and edema, were observed but resolved without special interventions.

**Conclusions:**

The combination of MFU and MFR for facial rejuvenation is both safe and effective. The combined treatment demonstrates superior enhancement in skin tightening, depigmentation, and pore refinement within a single therapeutic session.

## 1 Introduction

Skin aging is manifested by alterations in skin texture, decreased elasticity, and visible wrinkles [1, 2]. This issue not only impacts physical appearance but also has the potential to negatively affect mental well‐being [3, 4]. With the increasing aging trend of the population, there is a growing demand for facial rejuvenation procedures [5, 6, 7]. Therefore, the selection of treatment modalities that offer superior efficacy and minimal adverse effects holds clinical and social significance.

Clinical interventions for improving skin aging encompass a range of approaches, including surgery, laser therapy, chemical peels, and injections [8, 9, 10, 11, 12]. While surgery is often considered the preferred method for facial rejuvenation, it is important to note its limitations, including low patient tolerance, high surgical risks, and extended recovery periods [13, 14, 15]. In contrast, less invasive treatments such as laser therapy, chemical peels, and injections have shown limited efficacy in improving skin texture and tightening tissue [16, 17, 18, 19]. It is crucial to recognize that single treatment may not yield significant improvements in wrinkles or skin laxity.

In recent years, a novel non‐surgical approach to facial rejuvenation has emerged. The new micro‐focused ultrasound (MFU) device is capable of rapidly heating subcutaneous tissue to temperatures ranging from 50°C to 60°C, resulting in the creation of precise thermal injury zones (TIZs) measuring 2.5–3 mm^3^ within the deep dermis and superficial musculoaponeurotic system (SMAS) [20, 21, 22]. The ensuing wound healing cascade activates the degeneration and contraction of collagen fibers, leading to significant collagen remodeling [23]. Importantly, this innovative system is able to target deeper tissues without causing harm to the epidermis, thereby improving treatment comfort and minimizing the occurrence of adverse reactions.

The microneedle fractional radiofrequency (MFR) system delivers energy to target tissues and elevates the temperature of dermal collagen fibers to 55°C–65°C through a combination of microneedle mechanical stimulation and radiofrequency thermal effects [24, 25]. This process facilitates thermal remodeling and the generation of new collagen, ultimately reducing pigmentation and inflammatory reactions. The MFR device employed in this study incorporates innovative stratified technology to facilitate energy release at varying depths. The needle length can be adjusted from 0.5 to 3.5 mm, enabling precise thermal coagulation of specific layers [26]. This not only enhances efficacy but also minimizes pain and reduces recovery time.

Currently, studies have demonstrated the efficacy of MFU and MFR treatments in addressing skin aging [27, 28, 29, 30]. However, there is limited literature on the concurrent utilization of combined therapy. Consequently, our study seeks to assess the efficacy and safety of combining MFU and MFR treatments for facial rejuvenation in Asian populations.

## 2 Methods

### 2.1 Patients and Study Design

This study was randomized, prospective, split‐face, self‐controlled, and evaluator‐blinded. A total of 26 participants, comprising one male and 25 females aged between 30 and 60 years, who presented with facial soft tissue laxity and expressed a desire for rejuvenation treatment, were recruited from the Department of Dermatology at the First Affiliated Hospital of Chongqing Medical University. The study was conducted in accordance with the principles outlined in the Helsinki Declaration and was approved by the Ethics Committee of the First Affiliated Hospital of Chongqing Medical University (approval date: October 16, 2023; clinical trial ethic number: 2023‐448). All participants provided written informed consent for the trial. The exclusion criteria included individuals who had received facial and neck rejuvenation treatment within the past 6 months, undergone previous facial and neck surgery, had active systemic or local infections, had local skin diseases affecting wound healing, were pregnant or lactating, had metallic foreign bodies or fillers in the treatment area, had systemic diseases such as immune deficiency, diabetes, or lupus, had significant organ dysfunction, neurological or psychiatric disorders, or had a body mass index (BMI) exceeding 30 kg/m^2^.

Prior to initiating the study, the allocation of participants to the combined side and control side was determined using a random sequence generated in an Office 2021 spreadsheet. The combined side received a combination of MFU and MFR treatments, while the control side only received MFU treatment.

### 2.2 Treatment Protocols

Before treatment, the patient's facial skin was cleansed with water, followed by drawing facial marks to determine the treatment zones and exclude facial depressions and sites unsuitable for treatment. The ultrasound coupling agent was uniformly applied to the face after disinfection. The Micro‐Focused Ultrasound Treatment System (MFUS Pro, Hunan Peninsula Medical Technology Co. Ltd., China), equipped with D4.5, D3.0, and D2.0 transducers, was used. All participants were set to receive the highest level of the device without topical anesthesia to test the safety of the whole system. For the treatment of one side, the following parameters were applied: D4.5 Transducer: 6.63 W (level V), 10 Hz, with 4800 dots performed in 8 min on the lower face and the perioral and preauricular areas; D3.0 Transducer: 6.63 W, 10 Hz, with 5500 dots in 9 min on the lower face, submental area, and middle face; D2.0 Transducer: 6.63 W, 10 Hz, with 1500 dots in 3 min on the upper face of the selected control side exclusively. During the treatment, specific areas such as the ligaments, nasolabial folds, and perioral wrinkles were treated with personalized strengthening techniques. Following the MFU treatment, MFR was conducted on the designated side utilizing high‐frequency electrocautery therapy equipment (United, Shenzhen Peninsula Medical Group, China). After cleaning the patient's face again, compound lidocaine cream (Tongfang Pharmaceutical Group Co. Ltd., China) was applied to the combined side for local anesthesia for 60 min, followed by disinfection with 70% alcohol. The parameters for MFR treatment were set as outlined in Table 1. The patient was followed up twice at 1 and 3 months after the treatment.

**TABLE 1:** The treatment parameters of MFR.

Region	Layer	Power (W)	Pulse width (ms)	Depth of needle insertion (mm)
Fronto‐temporal region	Not stratified	8	100	1.2
Middle face	The first layer	6	60	0.8
	The second layer	8	100	1.5
Lower face	The first layer	6	60	1.5
	The second layer	8	100	2.5

### 2.3 Evaluation

#### 2.3.1 Objective Evaluation

##### 2.3.1.1 VISIA Parameters

Before treatment and at follow‐up visits, all patients underwent photography using the VISIA 6.0 Complexion Analysis System (Canfield Scientific Inc., USA). Images of the front and side of both cheeks were captured, with VISIA analyzing the presence of wrinkles, spots, pores, texture, and other photoaging parameters to generate a numerical score. A lower score was indicative of a lower prevalence of these skin imperfections.

##### 2.3.1.2 Ultrasound Examination

We utilized Wisonic (Shenzhen, China) to measure the thickness of the epidermis, dermis, and subcutaneous fat layers on both sides at specific reference points (Landmarks A–C). Landmark A, located at the midpoint of the forehead, was defined by the intersection of a vertical line extending from the midpoint of the pupil and a horizontal line across the middle of the forehead. Landmark B, situated at the masseter region, was identified by positioning the probe parallel to the mandible, with its midpoint at the intersection of the masseter and the mandible. Landmark C was designated at the midpoint of the cheek. Measurements were performed by the same technician, who was blinded to the treatment conditions, while the subject was seated. The technician was instructed to apply the probe gently to avoid compressing the skin.

#### 2.3.2 Subjective Evaluation

The clinical efficacy measurements included the physician and participant Global Aesthetic Improvement Scale (GAIS) and Wrinkle Severity Ranking Scale (WSRS). The GAIS is a 5‐point scale used to assess improvement at each follow‐up compared to the baseline photograph (Grade 0 = worse than the initial state in appearance; Grade 1 = no change; Grade 2 = improved; Grade 3 = much improved; Grade 4 = very much improved). The WSRS is a validated 5‐point scale used to evaluate wrinkle severity (Grade 0 = no wrinkles; Grade 1 = mild wrinkles; Grade 2 = moderate wrinkles; Grade 3 = severe wrinkles; Grade 4 = very severe wrinkles). All assessment criteria were conducted by two blinded dermatologists under consistent lighting and positioning. Scores were averaged in cases of discrepancies among evaluators. Standardized clinical photographs were captured using a professional digital camera (60D camera; Canon, Tokyo, Japan). Consistent lighting conditions, parameters, and patient positioning were maintained throughout the photography sessions, with photos taken before treatment and at 1 and 3 months post‐treatment. Patients were asked to complete a satisfaction questionnaire utilizing a 5‐point scale to indicate their level of contentment with the treatment, ranging from 1 (very satisfied) to 5 (very dissatisfied). Patient satisfaction with skin and facial appearance was also categorized as very satisfied, satisfied, neutral, or not satisfied.

### 2.4 Safety and Side Effects

During the MFU and MFR treatments, the severity of pain sensation was assessed using the Visual Analog Scale (VAS), with a range from 0 indicating no sensation to 10 representing the highest level of pain. Any potential side effects, such as pain, edema, erythema, oozing, purpura, post‐inflammatory hyperpigmentation, and scarring, were documented during the follow‐up period.

### 2.5 Statistical Analysis

Statistical analysis was conducted using SPSS 27.0 (IBM, New York, USA) and Origin 2021 (Origin Software Inc., California, USA). Normality of data was assessed using the Shapiro–Wilk test. A paired samples *t*‐test was employed for statistical analysis when two sets of dependent variables followed a normal distribution; otherwise, a paired samples Wilcoxon test was utilized. A significance level of *p* < 0.05 was considered statistically significant. Results for normally distributed data are presented as mean ± standard deviation, while non‐normally distributed data are represented as the median with a range.

## 3 Results

### 3.1 Demographic Characteristics

All 26 participants underwent a single treatment and completed the follow‐up examinations after 1 and 3 months. The demographic characteristics of the patients are presented in Table 2. The study included 25 female patients and 1 male patient. The mean age of the participants was 43.19 ± 7.58 years (range: 31–58 years), and the mean BMI of the patients was 21.44 ± 2.08 kg/m^2^ (range: 17.30–26.72 kg/m^2^). Of the participants, 18 were classified as Fitzpatrick skin type III and 8 as type IV.

**TABLE 2:** The demographic characteristics of the patients.

Demographic	Mean ± SD (median) (min–max) or *n* (%)
Age, years	43.19 ± 7.58 (41) (31–58)
BMI, kg/m^2^	21.44 ± 2.08 (21.48) (17.30–26.72)
Sex	
Female	25 (96.2%)
Male	1 (3.8%)
Fitzpatrick sun‐reactive skin type	
III	18 (69.2%)
IV	8 (30.8%)

Abbreviation: BMI, body mass index.

### 3.2 Objective Evaluation

#### 3.2.1 VISIA Parameters

**FIGURE 1:**
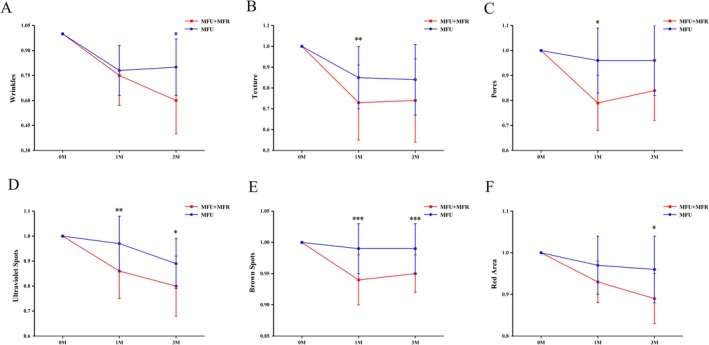
The VISIA scores of (A) wrinkles, (B) texture, (C) pores, (D) ultraviolet spots, (E) brown spots, and (F) red areas on the MFU + MFR side and MFU side (*p < 0.05; **p < 0.005; ***p < 0.001).

Eight variables were assessed in the analysis of skin complexion: spots, wrinkles, texture, pores, ultraviolet spots, brown spots, red areas, and porphyrins. Prior to initiating the treatment, baseline characteristics of both sides of the facial features were measured and found to be statistically similar (*p* > 0.05). From baseline to month 3, the scores of VISIA parameters on both sides exhibited a downward trend. The absolute scores of wrinkles, pores, ultraviolet spots, brown spots, and red area on the combined side demonstrated statistical significance at both 1 and 3 months post‐treatment when compared with pre‐treatment levels. On the control side, the absolute scores of wrinkles at 1 and 3 months, as well as the absolute score of texture at 1 month, were statistically significant compared to pre‐treatment levels (Supporting Information Table S1). At the first follow‐up, the average score of texture decreased by 27% on the combined side, whereas it decreased by 15% on the control side, indicating a significant improvement in texture on the combined side (*P*_between_ = 0.003). At the third month post‐treatment, there was a slight rebound on the combined side; however, a significant improvement in skin texture compared to baseline remained evident, as shown in Figure 1B. The average score of pores exhibited a 21% decrease on the combined side at the first month follow‐up, compared to a 4% decrease on the control side (*P*_between_ = 0.033). Although a rebound was observed at the third month follow‐up, a notable improvement compared to both baseline and the control side persisted, as depicted in Figure 1C. The average scores of wrinkles and red areas on the combined side decreased by 40% and 11%, respectively, at the second follow‐up, compared to reductions of 20% (*P*_between_ = 0.02) and 4% (*P*_between_ = 0.01) on the control side, demonstrating a statistically significant improvement, as illustrated in Figure 1A,F. For the average scores of ultraviolet spots and brown spots, statistical analysis revealed a significant disparity between the combined and control sides, indicating a greater reduction of spots on the combined side (*p* < 0.05), and the differences persisted until the third month (Figure 1D,E). Typical improvements in brown spots and ultraviolet spots after 3 months of combined treatment were shown in Supporting Information Figure S1.

#### 3.2.2 Ultrasound Examination

**FIGURE 2:**
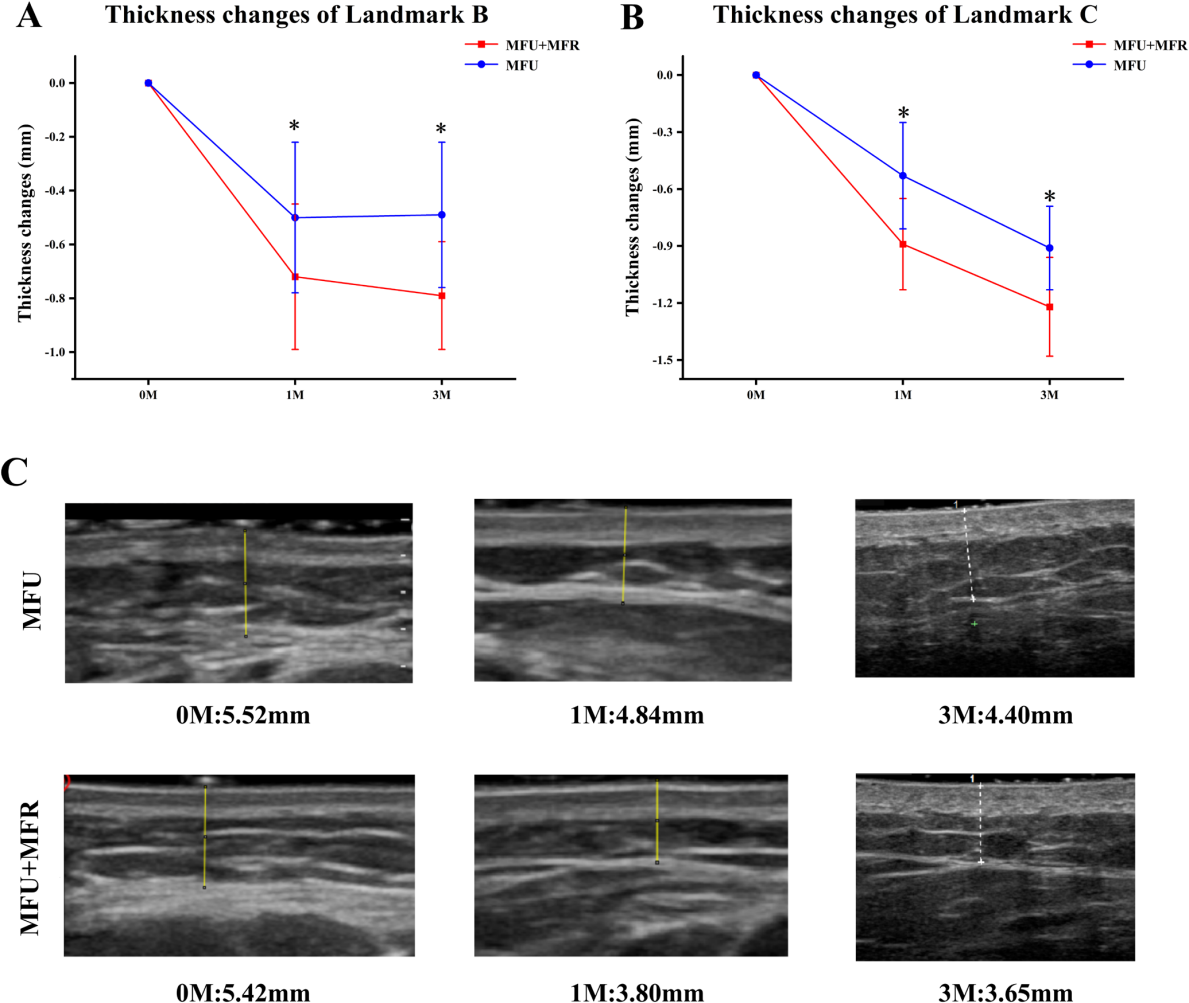
Thickness changes of (A) Landmark B and (B) Landmark C. (C) Typical thickness changes at the masseter of two sides before treatment and 1 to 3 months after treatment.

Before initiating the treatment, the baseline subcutaneous fat thickness was measured on both sides and found to be statistically similar (*p* > 0.05). The mean subcutaneous fat thickness at landmarks A, B, and C on both sides showed a significant reduction compared to baseline measurements. The mean subcutaneous fat thickness for landmarks A, B, and C on the combined side was 3.46 ± 0.68, 5.15 ± 0.87, and 6.92 ± 0.37 mm, respectively, before treatment. At the third month after treatment, these values decreased to 3.12 ± 0.60, 4.40 ± 0.81, and 5.58 ± 0.77 mm, respectively (Table 3). There was no significant difference in subcutaneous thickness changes at landmark A between both sides during the follow‐up periods, while the mean thickness changes at landmarks B and C on the combined side were statistically different compared to the control side during the two follow‐ups (*p* < 0.05) (Figure 2A,B). Figure 2C shows the typical changes in thickness before and after at 1 and 3 months on two sides.

**TABLE 3:** The subcutaneous fat thickness measured by ultrasound.

Location	Group	Baseline	Month 1	Month 3
The middle of forehead (Landmark A)	MFU + MFR	3.46 ± 0.68	3.22 ± 0.65*	3.12 ± 0.60***
	MFU	3.46 ± 0.72	3.22 ± 0.64***	3.26 ± 0.65***
Masseter (Landmark B)	MFU + MFR	5.15 ± 0.87	4.42 ± 0.75***	4.40 ± 0.81***
	MFU	4.97 ± 0.92	4.46 ± 0.83***	4.47 ± 0.91***
The middle of the cheek (Landmark C)	MFU + MFR	6.92 ± 0.37	6.06 ± 0.64***	5.58 ± 0.77***
	MFU	6.74 ± 0.38	6.20 ± 0.45***	5.73 ± 0.50***

*Note:* Compared with baseline: *p < 0.05; ***p < 0.001.

### 3.3 Subjective Evaluation

**FIGURE 3:**
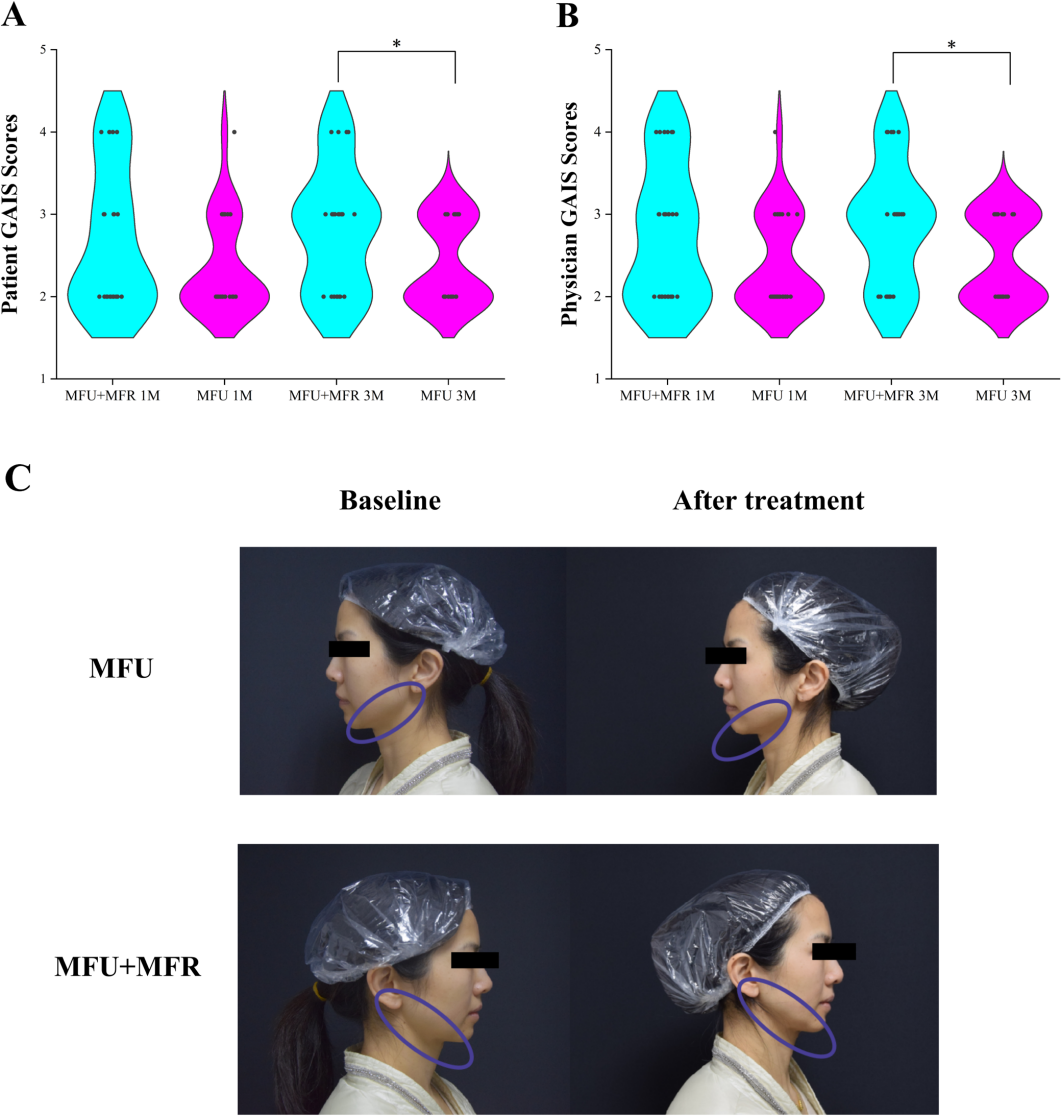
The violin graph of the GAIS scores of (A) patients and (B) physicians. (C) The improvements in skin tightness and jaw line clarity of a 43‐year‐old female patient.

Statistically significant improvements were observed in the GAIS and WSRS scores compared to baseline. The GAIS scores of the participants and the physicians improved after 1 and 3 months. Compared to the control side, the combined side showed statistically significant differences in GAIS scores at month 3 (Table 4 and Figure 3). At the second follow‐up, the GAIS scores of the participants and the physicians on the combined side were significantly increased to 2.95 ± 0.05 and 2.94 ± 0.07, respectively, compared to 2.66 ± 0.04 and 2.54 ± 0.04 (*p* < 0.05). At baseline, the mean WSRS score was 2.96 ± 0.73 for both sides. The WSRS scores on the combined side significantly decreased from baseline to 2.15 ± 0.56 and 2.11 ± 0.64 at 1 and 3 months, respectively (*p* < 0.05 at all follow‐up visits). The mean absolute change from baseline in WSRS score on the combined side at 1 and 3 months was −0.81 and −0.85, respectively, compared to −0.65 and −0.58 on the control side. The percentage of participants who were either “very satisfied” or “satisfied” consistently exceeded 90% throughout the study duration across all groups. However, two patients on the control side still maintained a neutral attitude toward the outcome of the therapy (Supporting Information Figure S2).

**TABLE 4:** The GAIS scores of the patients and physicians.

GAIS scores	Follow‐up	MFU + MFR	MFU	*p*
Patient	Month 1	2.77 ± 0.06	2.52 ± 0.03	> 0.05
	Month 3	2.95 ± 0.05	2.66 ± 0.04	0.047*
Physician	Month 1	2.66 ± 0.05	2.48 ± 0.02	> 0.05
	Month 3	2.94 ± 0.07	2.54 ± 0.04	0.028*

*Note:* Compared with baseline: *p < 0.05.

### 3.4 Safety and Side Effects

During the treatment, it was observed that MFR caused slightly stronger pain (mean score 3.29, range 1–6) compared to MFU (mean score 2.58, range 1–4). All the participants experienced slight erythema, purpura, or edema, along with a mild sensation of heat post‐treatment. Erythema usually subsided within a few hours after treatment, while swelling normally abated within 3–72 h without special interventions. In cases of significant erythema and swelling, intermittent cold or ice compresses were found to be effective in mitigating symptoms. Notably, no patients experienced scabbing, pruritus, red rash, hyperpigmentation, or hypopigmentation as adverse effects of the treatment.

## 4 Discussion

This study represents a novel approach by combining MFU and MFR for skin rejuvenation. The primary objective of this study was to evaluate the efficacy and safety of a combined therapy utilizing MFU and MFR in stratified anti‐aging interventions. Compared to using MFU therapy alone, the combination therapy yielded superior outcomes in reducing wrinkles and improving skin texture and significantly enhancing the treatment of skin pigmentation, enlarged pores, and subcutaneous fat.

Our research demonstrates that the use of MFU as a standalone intervention effectively elevates and firms facial skin, corroborating previous findings in the field [31, 32]. The absolute scores for wrinkles and texture on the control side improved significantly compared to baseline. Ultrasound results revealed a significant reduction in the thickness of subcutaneous fat on the control side, aligning with prior studies [33]. The MFU device employed in our study offers treatment options at depths of 4.5, 3.0, and 2.0 mm. The MFU transducer at the depth of 4.5 mm effectively and precisely delivers energy to the SMAS, a complex fibro‐fat layer composed of collagen and elastic fibers interspersed among fat cells, inducing immediate collagen contraction and promoting subsequent collagen regeneration and remodeling [34]. The MFU transducer at the depth of 3.0 mm primarily targets the melting of subcutaneous adipose tissue, resulting in the final phagocytosis of decomposed fat cell contents by macrophages [35]. The MFU transducer at the depth of 2.0 mm acts on the dermis, which can stimulate collagen regeneration, tighten fine lines, and shrink pore and delicate skin [28]. Overall, MFU demonstrates significant potential for non‐invasive skin tightening and wrinkle improvement, offering promising prospects for facial rejuvenation.

MFR, known for its safety profile, minimal side effects, and short recovery period, has shown positive outcomes in anti‐aging treatments. Numerous studies provide evidence that MFR can effectively reduce enlarged pores [36], control inflammation [37] and diminish wrinkles [38]. Consistently, our study showed that the absolute scores of texture, pores, red area, and wrinkles on the combined side were significantly decreased, and the improvement ratios surpassed the control side with statistically significant difference. MFR has the capacity to induce the production of collagen and elastin, reinforce the integrity of pores, and impair the functionality of sebaceous glands, resulting in reduced sebum secretion and ultimately leading to pore constriction and less inflammation [39]. Compared to treatment with MFU alone, the changes in thickness of subcutaneous fat on the combined side were statistically significant during the two follow‐ups. MFR is capable of heating and coagulating the subcutaneous tissue, including dermis, fascia, and fat layer, depending on the depth of insertion [40]. The novel MFR system used in this study allows for layered heating as the needle is withdrawn, unlike traditional insulating needles that can only release energy at 0.3 mm. With disordered scanning technology and customized power and pulse width for each layer, the outcomes were transformed from two‐dimensional to three‐dimensional. This process stimulates collagen regeneration, fascia contraction, and fat lipolysis, resulting in skin rejuvenation and a more defined facial contour [41].

We unexpectedly discovered that the combination of MFU and MFR improves skin depigmentation through a synergistic effect. The results indicated a lasting and more pronounced reduction of ultraviolet spots and brown spots on the combined side compared with being treated by MFU alone. Previous studies have demonstrated the promising clinical effect of MFR in the treatment of melasma [42]. Microneedles can stimulate dermal tissue and reconstruct the structure of the dermis, facilitating the regulation of pigment granule metabolism and vascular function [43]. Additionally, this combination promotes collagen production in local skin tissue and enhances skin barrier function, creating a foundation for diminishing pigment fragments [44]. The effect of MFU on pigmentation observed in this study aligns with previous findings. This may be attributed to the vibrations and friction generated by ultrasound propagation, which can mechanically disrupt or eliminate melanin and pigment fragments above the TIZs [45, 46, 47]. The pigments and pigment fragments produced during this process may recruit macrophages, triggering a response similar to that seen in laser tattoo removal, thereby promoting pigment and epidermal metabolism [44]. Our study further demonstrated that the combined therapy resulted in a more pronounced reduction of fat pads, likely due to synergistic effects. MFU targets ultrasonic energy at the subcutaneous fat layers without damaging the epidermis, thereby promoting the breakdown and metabolism of fat cells [48, 49, 50]. MFR, on the other hand, delivers radiofrequency energy directly to the deeper layers through microneedles, effectively stimulating collagen regeneration and skin firmness [51, 52]. This technique not only improves skin texture and reduces sagging but also promotes fat cell breakdown and volume redistribution through thermal effects, thereby optimizing facial contours [53, 54]. When combined with MFR, this approach creates a synergistic effect that more effectively reduces fat deposits and enhances facial contour reshaping.

The significant improvements observed in the VISIA parameters in this study have substantial clinical implications for facial rejuvenation. The reduction in wrinkles and the improvement in skin texture on the combined side reflect enhancements in skin smoothness and firmness. These improvements are corroborated by significant enhancements in GAIS and WSRS scores compared to baseline. Additionally, the decrease in UV spots and brown spots, which are often the result of UV damage and aging, indicates effective pigmentation management. Furthermore, over 90% of participants reported being “very satisfied” or “satisfied” throughout the study, underscoring the clinical relevance of the observed improvements in VISIA parameters.

Given that both MFU and MFR rely on thermal stimulation for their efficacy, we opted to schedule these two treatments in a specific order on the same day to minimize the risk of significant thermal damage and subsequent adverse reactions. In regard to safety and comfort, no intramuscular anesthetics or painkillers were needed, while there were comparatively lower VAS scores of MFU and MFR. Our results indicated that this arrangement did not impact treatment outcomes or cause any serious side effects. The single pulse of the novel MFU device was comparably short, with a pulse width of less than 50 ms [55]. This extremely short pulse duration could reduce pain sensation and minimize side effects. By concentrating its energy at the tip, the novel MFR device effectively reduces the likelihood of epidermal involvement and mitigates associated risks. Additionally, it utilizes disordered scanning technology, which involves two needles in groups releasing energy simultaneously, resulting in increased energy strength and reduced pain in the treatment region. The upgraded MFR outputs high‐frequency current with ultra‐short pulse sequences, allowing for rapid energy transmission that effectively mitigates skin pain by preempting signal transmission to the brain.

We argue that a multilayered approach to anti‐aging is both safe and effective. It is our belief that the simultaneous application of anti‐aging treatments targeting diverse skin layers can lead to more effective rejuvenation. The validation of this claim could be supported by relatively lower VAS scores, higher GAIS scores of both patients and physicians, as well as a higher satisfaction rate reported by patients treated with combined therapy. Furthermore, the positive outcomes of the combined therapy persisted for 3 months post‐treatment, indicating a lasting rejuvenation effect. The utilization of combined therapy offers a comfortable and effective treatment approach.

However, this study has some limitations. First, the study included a small patient cohort and lacked long‐term follow‐up. The efficacy of a singular treatment remains inconclusive, necessitating multiple treatments. Future studies with larger sample sizes and prolonged follow‐up periods should be conducted to validate our results and further ascertain the long‐term effects of the novel MFU and MFR. Second, there was an imbalance in the participants' gender distribution, with 25 out of 26 participants being female. We plan to assess the effectiveness of these technologies in male subjects in future studies. Third, although ultrasound imaging and digital assessments were used to evaluate contour changes, potential bias may have arisen from micro‐expression changes and subtle head movements. More precise equipment should be adopted in the future to accurately evaluate subtle changes that may be difficult to distinguish by the investigator.

## 5 Conclusions

Our study investigated the safety and effectiveness of MFU combined with MFR for anti‐aging treatment across different facial layers in a single treatment. The combined approach is deemed highly safe with comparatively low pain and has demonstrated enhanced effects in skin tightening, pigment reduction, and pore refinement.

## Author Contributions

Ruiyao Wang, Guangling Peng, Yangmei Chen, and Jin Chen contributed to the study's conception and design. Ruiyao Wang and Guangling Peng contributed to the acquisition of data and writing of the initial article. Ruiyao Wang, Yangmei Chen, Jin Chen, and Lin Liu performed the statistical analyses and interpretation of data. Xinyi Shao, Tingqiao Chen, Megcen Shi, Judan Zhong, and Yi Ou provided editing support. All authors contributed to the interpretation and analysis of the literature, as well as careful and critical revision and approval of the final manuscript.

## Ethics Statement

The present study was approved by the Ethics Committee of the First Affiliated Hospital of Chongqing Medical University (approval date: October 16, 2023; clinical trial ethic number: 2023‐448). Written informed consent was obtained from each participant before enrollment, and this study adhered to the Principle of the Declaration of Helsinki.

## Conflicts of Interest

The authors declare no conflicts of interest.

## Supporting information


**Data S1:** jocd70455‐sup‐0001‐FigureS1.tif.

**Data S2:** jocd70455‐sup‐0002‐FigureS2.tif.

**Data S3:** jocd70455‐sup‐0003‐TableS1.docx.

## Data Availability

The datasets used during the current study are available from the corresponding author upon reasonable request.

## References

[1] E. Kohl , J. Steinbauer , M. Landthaler , and R. M. Szeimies , “Skin Ageing,” Journal of the European Academy of Dermatology and Venereology 25, no. 8 (2011): 873–884, 10.1111/j.1468-3083.2010.03963.x. 21261751

[2] F. Bonté , D. Girard , J. C. Archambault , and A. Desmoulière , “Skin Changes During Ageing,” Sub‐Cellular Biochemistry 91 (2019): 249–280, 10.1007/978-981-13-3681-2_10. 30888656

[3] T. M. Ansary , M. R. Hossain , K. Kamiya , M. Komine , and M. Ohtsuki , “Inflammatory Molecules Associated With Ultraviolet Radiation‐Mediated Skin Aging,” International Journal of Molecular Sciences 22, no. 8 (2021): 3974, 10.3390/ijms22083974. 33921444 PMC8069861

[4] M. Chaudhary , A. Khan , and M. Gupta , “Skin Ageing: Pathophysiology and Current Market Treatment Approaches,” Current Aging Science 13, no. 1 (2020): 22–30, 10.2174/1567205016666190809161115. 31530270 PMC7403684

[5] T. W. Griffiths , R. E. B. Watson , and A. K. Langton , “Skin Ageing and Topical Rejuvenation Strategies,” British Journal of Dermatology 189, no. Suppl 1 (2023): i17–i23, 10.1093/bjd/ljad282. 37903073

[6] F. G. Fedok and J. G. Lighthall , “Evaluation and Treatment Planning for the Aging Face Patient,” Clinics in Plastic Surgery 50, no. 3 (2023): 367–380, 10.1016/j.cps.2022.12.002. 37169403

[7] S. Zhang and E. Duan , “Fighting Against Skin Aging: The Way From Bench to Bedside,” Cell Transplantation 27, no. 5 (2018): 729–738, 10.1177/0963689717725755. 29692196 PMC6047276

[8] J. Zhu , X. Ji , Y. Xu , et al., “The Efficacy of Intradermal Injection of Type A Botulinum Toxin for Facial Rejuvenation,” Dermatologic Therapy 30, no. 1 (2017): e12433, 10.1111/dth.12433. 27862727

[9] S. Yildirim , M. S. Gurel , S. Gungor , O. Tekeli , and D. Canat , “Comparison of Efficacy of Chemical Peeling With 25% Trichloroacetic Acid and 0.1% Retinoic Acid for Facial Rejuvenation,” Postępy Dermatologii I Alergologii 33, no. 3 (2016): 199–205, 10.5114/ada.2016.60612. 27512355 PMC4969415

[10] M. El‐Domyati , T. Abd‐El‐Raheem , W. Medhat , H. Abdel‐Wahab , and M. Al Anwer , “Multiple Fractional Erbium: Yttrium‐Aluminum‐Garnet Laser Sessions for Upper Facial Rejuvenation: Clinical and Histological Implications and Expectations,” Journal of Cosmetic Dermatology 13, no. 1 (2014): 30–37, 10.1111/jocd.12079. 24641603

[11] M. Raffaini , A. Magri , M. Conti , and F. Arcuri , “Advanced Facial Rejuvenation After Bimaxillary Surgery in Three Different Facial Types,” Aesthetic Plastic Surgery 46, no. 1 (2022): 183–193, 10.1007/s00266-021-02399-w. 34131790

[12] Z. Li , H. Wu , Z. Yang , et al., “Combining Liposuction and Thread‐Lifting for Middle‐Lower Facial Rejuvenation,” Aesthetic Plastic Surgery 48, no. 9 (2024): 1672–1678, 10.1007/s00266-024-03872-y. 38448603

[13] I. Pitanguy and B. H. Machado , “Facial Rejuvenation Surgery: A Retrospective Study of 8788 Cases,” Aesthetic Surgery Journal 32, no. 4 (2012): 393–412, 10.1177/1090820x12438895. 22375057

[14] B. Azizzadeh , R. Fitzgerald , G. Massry , and E. Smith , “Subunit Approach to Facelifting and Facial Rejuvenation,” Facial Plastic Surgery Clinics of North America 28, no. 3 (2020): 253–272, 10.1016/j.fsc.2020.03.004. 32503713

[15] P. K. Sullivan , E. A. Hoy , V. Mehan , and D. P. Singer , “An Anatomical Evaluation and Surgical Approach to the Perioral Mound in Facial Rejuvenation,” Plastic and Reconstructive Surgery 126, no. 4 (2010): 1333–1340, 10.1097/PRS.0b013e3181ea4abd. 20885255

[16] M. I. Rendon , D. S. Berson , J. L. Cohen , W. E. Roberts , I. Starker , and B. Wang , “Evidence and Considerations in the Application of Chemical Peels in Skin Disorders and Aesthetic Resurfacing,” Journal of Clinical and Aesthetic Dermatology 3, no. 7 (2010): 32–43. PMC2921757 20725555

[17] J. D. Meaike , N. Agrawal , D. Chang , E. I. Lee , and M. G. Nigro , “Noninvasive Facial Rejuvenation. Part 3: Physician‐Directed‐Lasers, Chemical Peels, and Other Noninvasive Modalities,” Seminars in Plastic Surgery 30, no. 3 (2016): 143–150, 10.1055/s-0036-1584818. 27478423 PMC4961506

[18] J. Fanning , C. Jacob , I. Diaz , and O. Ibrahim , “Treatment of Mild‐To‐Moderate Facial Cutaneous Aging Using a Combination Peel Containing 6% Trichloroacetic Acid and 12% Lactic Acid,” Journal of Cosmetic Dermatology 22, no. 11 (2023): 3033–3041, 10.1111/jocd.15814. 37227430

[19] Y. Gronovich , Y. Raderman , R. Toledano , et al., “Evaluation of a Novel Ablative 1940 nm Pulsed Laser for Skin Rejuvenation,” Lasers in Surgery and Medicine 56 (2024): 592–596, 10.1002/lsm.23817. 38890781

[20] D. H. Suh , M. K. Shin , S. J. Lee , et al., “Intense Focused Ultrasound Tightening in Asian Skin: Clinical and Pathologic Results,” Dermatologic Surgery 37, no. 11 (2011): 1595–1602, 10.1111/j.1524-4725.2011.02094.x. 21806707

[21] G. Oni , R. Hoxworth , S. Teotia , S. Brown , and J. M. Kenkel , “Evaluation of a Microfocused Ultrasound System for Improving Skin Laxity and Tightening in the Lower Face,” Aesthetic Surgery Journal 34, no. 7 (2014): 1099–1110, 10.1177/1090820x14541956. 24990884

[22] H. Hugul , M. C. Oba , M. Kirisci , and Z. Kutlubay , “Focused Radiofrequency and Ultrasound for Face and Neck Rejuvenation: A Retrospective Evaluation of 158 Patients,” Journal of Cosmetic Dermatology 21, no. 1 (2022): 290–295, 10.1111/jocd.14510. 34601819

[23] W. M. White , I. R. Makin , P. G. Barthe , M. H. Slayton , and R. E. Gliklich , “Selective Creation of Thermal Injury Zones in the Superficial Musculoaponeurotic System Using Intense Ultrasound Therapy: A New Target for Noninvasive Facial Rejuvenation,” Archives of Facial Plastic Surgery 9, no. 1 (2007): 22–29, 10.1001/archfaci.9.1.22. 17224484

[24] B. M. Hantash , B. Renton , R. L. Berkowitz , B. C. Stridde , and J. Newman , “Pilot Clinical Study of a Novel Minimally Invasive Bipolar Microneedle Radiofrequency Device,” Lasers in Surgery and Medicine 41, no. 2 (2009): 87–95, 10.1002/lsm.20687. 19226570

[25] J. Feng , L. Zhang , J. Qi , and L. Huang , “Histological Damage Characteristics and Quantitive Analysis of Porcine Skin With Non‐Insulated Microneedle Radiofrequency,” Skin Research and Technology 29, no. 6 (2023): e13396, 10.1111/srt.13396. 37357651 PMC10264746

[26] Y. Liu , W. Yu , J. Zhu , et al., “Facial Tightening Using a Novel Vacuum‐Assisted Microneedle Fractional Radiofrequency System: A Prospective, Randomized, Split‐Face Study,” Journal of Cosmetic Dermatology 23 (2024): 3248–3255, 10.1111/jocd.16414. 38923679 PMC13649113

[27] B. Yalici‐Armagan and G. Elcin , “Evaluation of Microfocused Ultrasound for Improving Skin Laxity in the Lower Face: A Retrospective Study,” Dermatologic Therapy 33, no. 6 (2020): e14132, 10.1111/dth.14132. 32770566

[28] W. Chen , Y. Deng , G. Qiao , and W. Cai , “Ultrasound Rejuvenation for Upper Facial Skin: A Randomized Blinded Prospective Study,” Journal of Cosmetic Dermatology 23 (2024): 3942–3949, 10.1111/jocd.16482. 39034504 PMC11626351

[29] W. Dou , Q. Yang , Y. Yin , et al., “Fractional Microneedle Radiofrequency Device and Fractional Erbium‐Doped Glass 1,565‐nm Device Treatment of Human Facial Photoaging: A Prospective, Split‐Face, Random Clinical Trial,” Journal of Cosmetic and Laser Therapy 23, no. 5–6 (2021): 142–148, 10.1080/14764172.2022.2033783. 35083965

[30] E. A. Spataro , K. Dierks , and P. J. Carniol , “Microneedling‐Associated Procedures to Enhance Facial Rejuvenation,” Facial Plastic Surgery Clinics of North America 30, no. 3 (2022): 389–397, 10.1016/j.fsc.2022.03.012. 35934440

[31] D. Shome , S. Vadera , M. S. Ram , S. Khare , and R. Kapoor , “Use of Micro‐Focused Ultrasound for Skin Tightening of Mid and Lower Face,” Plastic and Reconstructive Surgery. Global Open 7, no. 12 (2019): e2498, 10.1097/gox.0000000000002498. 32537285 PMC7288869

[32] U. Khan and N. Khalid , “A Systematic Review of the Clinical Efficacy of Micro‐Focused Ultrasound Treatment for Skin Rejuvenation and Tightening,” Cureus 13, no. 12 (2021): e20163, 10.7759/cureus.20163. 35003992 PMC8722640

[33] J. Zhu , Y. Han , Y. Liu , et al., “Evaluation of a Novel Microfocused Ultrasound With Three‐Dimensional Digital Imaging for Facial Tightening: A Prospective, Randomized, Controlled Trial,” Dermatology and Therapy (Heidelberg) 14, no. 1 (2024): 233–249, 10.1007/s13555-023-01078-9. PMC11756818 38100073

[34] J. Ling and H. Zhao , “A Systematic Review and Meta‐Analysis of the Clinical Efficacy and Patients' Satisfaction of Micro‐Focused Ultrasound (MFU) Treatment for Facial Rejuvenation and Tightening,” Aesthetic Plastic Surgery 47, no. 5 (2023): 1806–1823, 10.1007/s00266-023-03384-1. 37198297

[35] M. L. Jewell , C. Desilets , and B. R. Smoller , “Evaluation of a Novel High‐Intensity Focused Ultrasound Device: Preclinical Studies in a Porcine Model,” Aesthetic Surgery Journal 31, no. 4 (2011): 429–434, 10.1177/1090820x11405026. 21551434

[36] Y. Huang , H. Zheng , Q. Wu , and M. Zhang , “Efficacy and Safety of Fractional Micro‐Needling Radiofrequency for the Treatment of Enlarged Pores on the Cheeks of a Chinese Cohort: A Retrospective Study,” Lasers in Medical Science 39, no. 1 (2024): 100, 10.1007/s10103-024-04043-w. 38609686

[37] L. Huang , Y. Liu , W. Fang , et al., “Efficiency and Safety of Microneedling Fractional Radiofrequency in the Treatment of Chinese Atrophic Acne Scars: A Retrospective Study of 3 Consecutive Treatments With 1‐Month Intervals,” Journal of Cosmetic Dermatology 22, no. 2 (2023): 497–504, 10.1111/jocd.15454. 36217740

[38] Y. Gao , Q. Che , Q. He , et al., “Treatment of Periorbital Aging With Negative Pressure Fractional Microneedle Radiofrequency: A Self‐Controlled Clinical Trial,” Journal of Cosmetic Dermatology 23, no. 4 (2024): 1269–1276, 10.1111/jocd.16091. 38013586

[39] S. I. Cho , B. Y. Chung , M. G. Choi , et al., “Evaluation of the Clinical Efficacy of Fractional Radiofrequency Microneedle Treatment in Acne Scars and Large Facial Pores,” Dermatologic Surgery 38, no. 7 Pt 1 (2012): 1017–1024, 10.1111/j.1524-4725.2012.02402.x. 22487513

[40] K. Y. Seo , M. S. Yoon , D. H. Kim , and H. J. Lee , “Skin Rejuvenation by Microneedle Fractional Radiofrequency Treatment in Asian Skin; Clinical and Histological Analysis,” Lasers in Surgery and Medicine 44, no. 8 (2012): 631–636, 10.1002/lsm.22071. 22936274

[41] R. M. Javate , R. T. Cruz, Jr. , J. Khan , N. Trakos , and R. E. Gordon , “Nonablative 4‐MHz Dual Radiofrequency Wand Rejuvenation Treatment for Periorbital Rhytides and Midface Laxity,” Ophthalmic Plastic and Reconstructive Surgery 27, no. 3 (2011): 180–185, 10.1097/IOP.0b013e3181fe8e5a. 21283035

[42] H. J. Han , J. C. Kim , Y. J. Park , and H. Y. Kang , “Targeting the Dermis for Melasma Maintenance Treatment,” Scientific Reports 14, no. 1 (2024): 949, 10.1038/s41598-023-51133-w. 38200171 PMC10781782

[43] X. Wu , Z. Zhang , J. Zhu , et al., “Can Microneedle Fractional Radiofrequency System Treatment Impair the Skin Barrier Function in Chinese Patients? A Prospective Clinical Trial,” Dermatology and Therapy (Heidelberg) 12, no. 10 (2022): 2371–2382, 10.1007/s13555-022-00807-w. PMC9515261 36129670

[44] N. Pazyar , M. Raeispour , R. Yaghoobi , and M. Seyedtabib , “Evaluation of the Effectiveness of Microneedling With Tranexamic Acid in Comparison With Microneedling With Vitamin C in the Treatment of Melasma: A Prospective and Single‐Blind Clinical Trial,” Health Science Reports 6, no. 10 (2023): e1636, 10.1002/hsr2.1636. 37867785 PMC10588557

[45] V. Vachiramon , N. Jurairattanaporn , S. Harnchoowong , and P. Chayavichitsilp , “Non‐Invasive High‐Intensity Focused Ultrasound for UV‐Induced Hyperpigmentation in Fitzpatrick Skin Types III and IV: A Prospective, Randomized, Controlled, Evaluator‐Blinded Trial,” Lasers in Medical Science 33, no. 2 (2018): 361–367, 10.1007/s10103-017-2393-5. 29177714

[46] J. T. E. Lim , “Safety and Efficacy of Superficial Micro‐Focused Ultrasound With Visualization for Melasma in Asians: An Uncontrolled Pilot Study,” Journal of Cosmetic Dermatology 22, no. 6 (2023): 1764–1773, 10.1111/jocd.15661. 36762392

[47] S. Y. Choi , K. H. Yoo , C. T. Oh , et al., “High Intensity Focused Ultrasound as a Potential New Modality for the Treatment of Pigmentary Skin Disorder,” Skin Research and Technology 22, no. 2 (2016): 131–136, 10.1111/srt.12239. 26094501

[48] D. Duncan , “Megasessions: Efficacy of Fewer, Longer Treatment Sessions for Fat Reduction in Noninvasive Body Contouring Using a Radiofrequency Based Device,” Journal of Drugs in Dermatology 16, no. 5 (2017): 478–480. 28628684

[49] M. A. Adatto , R. M. Adatto‐Neilson , and G. Morren , “Reduction in Adipose Tissue Volume Using a New High‐Power Radiofrequency Technology Combined With Infrared Light and Mechanical Manipulation for Body Contouring,” Lasers in Medical Science 29, no. 5 (2014): 1627–1631, 10.1007/s10103-014-1564-x. 24687404 PMC4149887

[50] E. J. Ko , H. Kim , W. S. Park , and B. J. Kim , “Correction of Midface Volume Deficiency Using Hyaluronic Acid Filler and Intradermal Radiofrequency,” Journal of Cosmetic and Laser Therapy 17, no. 1 (2015): 46–48, 10.3109/14764172.2014.968579. 25264587

[51] H. J. Lee , S. R. Seo , M. S. Yoon , J. Y. Song , E. Y. Lee , and S. E. Lee , “Microneedle Fractional Radiofrequency Increases Epidermal Hyaluronan and Reverses Age‐Related Epidermal Dysfunction,” Lasers in Surgery and Medicine 48, no. 2 (2016): 140–149, 10.1002/lsm.22420. 26415023

[52] H. Kaplan and L. Kaplan , “Combination of Microneedle Radiofrequency (RF), Fractional RF Skin Resurfacing and Multi‐Source Non‐Ablative Skin Tightening for Minimal‐Downtime, Full‐Face Skin Rejuvenation,” Journal of Cosmetic and Laser Therapy 18, no. 8 (2016): 438–441, 10.1080/14764172.2016.1228981. 27594273

[53] L. Nguyen , J. Bartholomeusz , S. W. Schneider , and K. Herberger , “Histological and Clinical Dose‐Response Analysis of Radiofrequency Microneedling Treatment for Skin Rejuvenation,” Lasers in Medical Science 40, no. 1 (2025): 75, 10.1007/s10103-025-04335-9. 39915343 PMC11802595

[54] D. Hurwitz and D. Smith , “Treatment of Overweight Patients by Radiofrequency‐Assisted Liposuction (RFAL) for Aesthetic Reshaping and Skin Tightening,” Aesthetic Plastic Surgery 36, no. 1 (2012): 62–71, 10.1007/s00266-011-9783-z. 21751063

[55] W. Franco , A. Kothare , S. J. Ronan , R. C. Grekin , and T. H. McCalmont , “Hyperthermic Injury to Adipocyte Cells by Selective Heating of Subcutaneous Fat With a Novel Radiofrequency Device: Feasibility Studies,” Lasers in Surgery and Medicine 42, no. 5 (2010): 361–370, 10.1002/lsm.20925. 20583242

