# Supplementary figures and images for: Combined Novel Microfocused Ultrasound and Microneedle Fractional Radiofrequency System for Multilayered Facial Rejuvenation: A Prospective, Randomized, and Split‐Face Study
Source: J Cosmet Dermatol. 2025 Sep 22;24(10):e70455. doi: 10.1111/jocd.70455 (PMC12452053; doi:10.1111/jocd.70455)

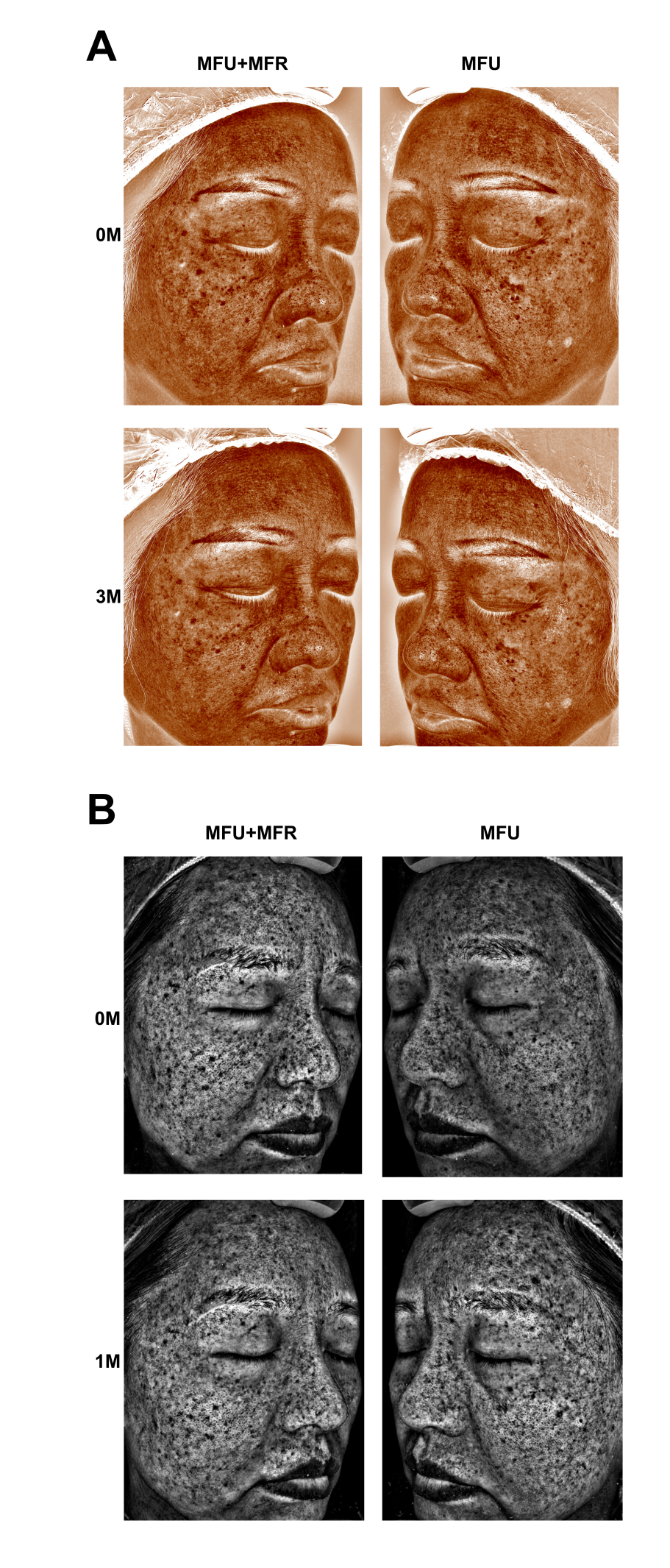

Supplement: Supplementary file 1 — Data S1: jocd70455‐sup‐0001‐FigureS1.tif. [file JOCD-24-e70455-s003.tif]
