# Supplementary material for: Combined Novel Microfocused Ultrasound and Microneedle Fractional Radiofrequency System for Multilayered Facial Rejuvenation: A Prospective, Randomized, and Split‐Face Study
Source: J Cosmet Dermatol. 2025 Sep 22;24(10):e70455. doi: 10.1111/jocd.70455 (PMC12452053; doi:10.1111/jocd.70455)
Supplement: Supplementary file 3 — Data S3: jocd70455‐sup‐0003‐TableS1.docx. [file JOCD-24-e70455-s001.docx]

| **Parameter** | **Group** | **Baseline** | **Month 1** | **Month 3** |
| --- | --- | --- | --- | --- |
| Spots | MFU + MFR | 34.34±6.39 | 29.96±6.40******* | 29.11±5.34******* |
|  | MFU | 31.41±6.01 | 30.58±6.68 | 31.28±6.06 |
| Wrinkle | MFU + MFR | 11.20(4.64,23.53) | 6.58(3.27,19.00)******* | 7.38(2.90,13.81)******* |
|  | MFU | 9.59(4.69,17.31) | 5.26(1.75,13.59)***** | 9.28(3.67,15.89) |
| Texture | MFU + MFR | 10.86(6.17,20.28) | 8.34(4.00,15.13)******* | 7.40(5.36,14.72)******* |
|  | MFU | 11.00(6.52,17.38) | 9.35(4.67,15.32)******* | 10.73(5.14,14.72)******* |
| Pores | MFU + MFR | 10.86(6.17,20.28) | 8.37(4.06,15.13)******* | 9.49(5.85,12.56)****** |
|  | MFU | 11.00(17.37,6.52) | 10.85(5.44,15.75) | 10.73(5.14,16.03) |
| Ultraviolet Spots | MFU + MFR | 16.71(12.19,26.86) | 13.32(10.26,21.80)****** | 12.29(9.42,18.68)******* |
|  | MFU | 15.80(11.94,20.53) | 15.16(11.40,20.51) | 13.78(11.18,19.67) |
| Brown Spots | MFU + MFR | 39.84(36.99,41.91) | 37.91(34.83,40.72)****** | 37.94(35.55,40.44)******* |
|  | MFU | 39.14(34.43,41.99) | 38.90(34.71,41.58) | 38.70(35.50,41.59) |
| Red Areas | MFU + MFR | 27.64±4.02 | 25.78±3.43******* | 24.77±4.30******* |
|  | MFU | 27.05±3.95 | 26.20±3.00 | 26.09±3.80 |
| Porphyrins | MFU + MFR | 3.87(2.20,6.23) | 5.11(3.68,7.21)****** | 2.90(1.66,5.06) |
|  | MFU | 4.30(2.12,6.67) | 5.18(3.21,6.47) | 3.01(1.77,5.02) |

Supplementary Table 1 The scores of VISIA parameters.

(Compared with baseline: *, P <0.05; **, P < 0.005; ***, P < 0.001)
